# Determinants of acute kidney injury during high-power mechanical ventilation: secondary analysis from experimental data

**DOI:** 10.1186/s40635-024-00610-1

**Published:** 2024-03-21

**Authors:** Simone Gattarello, Fabio Lombardo, Federica Romitti, Rosanna D’Albo, Mara Velati, Isabella Fratti, Tommaso Pozzi, Rosmery Nicolardi, Antonio Fioccola, Mattia Busana, Francesca Collino, Peter Herrmann, Luigi Camporota, Michael Quintel, Onnen Moerer, Leif Saager, Konrad Meissner, Luciano Gattinoni

**Affiliations:** 1 Department of Anesthesia and Intensive Care Medicine Department, IRCCS San Raffaele Scientific Institute, Via Olgettina 60, 20132 Milan, Italy; 2 Department of Anesthesiology, University Medical Centre Göttingen, Göttingen, Germany; 3 Department of Anesthesia, Intensive Care and Emergency, “Città Della Salute E Della Scienza” Hospital, Turin, Italy; 4 Department of Adult Critical Care, Guy’s and St. Thomas’ NHS Foundation Trust, London, UK; 5 Department of Anesthesiology, Intensive Care and Emergency Medicine Donau-Isar-Klinikum Deggendorf, Deggendorf, Germany

**Keywords:** Mean perfusion pressure, Mean arterial pressure, Central venous pressure, Acute kidney injury, Acute kidney failure, Mechanical power, Mechanical ventilation

## Abstract

**Background:**

The individual components of mechanical ventilation may have distinct effects on kidney perfusion and on the risk of developing acute kidney injury; we aimed to explore ventilatory predictors of acute kidney failure and the hemodynamic changes consequent to experimental high-power mechanical ventilation.

**Methods:**

Secondary analysis of two animal studies focused on the outcomes of different mechanical power settings, including 78 pigs mechanically ventilated with high mechanical power for 48 h. The animals were categorized in four groups in accordance with the RIFLE criteria for acute kidney injury (AKI), using the end-experimental creatinine: (1) NO AKI: no increase in creatinine; (2) RIFLE 1-Risk: increase of creatinine of > 50%; (3) RIFLE 2-Injury: two-fold increase of creatinine; (4) RIFLE 3-Failure: three-fold increase of creatinine;

**Results:**

The main ventilatory parameter associated with AKI was the positive end-expiratory pressure (PEEP) component of mechanical power. At 30 min from the initiation of high mechanical power ventilation, the heart rate and the pulmonary artery pressure progressively increased from group NO AKI to group RIFLE 3. At 48 h, the hemodynamic variables associated with AKI were the heart rate, cardiac output, mean perfusion pressure (the difference between mean arterial and central venous pressures) and central venous pressure. Linear regression and receiving operator characteristic analyses showed that PEEP-induced changes in mean perfusion pressure (mainly due to an increase in CVP) had the strongest association with AKI.

**Conclusions:**

In an experimental setting of ventilation with high mechanical power, higher PEEP had the strongest association with AKI. The most likely physiological determinant of AKI was an increase of pleural pressure and CVP with reduced mean perfusion pressure. These changes resulted from PEEP per se and from increase in fluid administration to compensate for hemodynamic impairment consequent to high PEEP;

**Supplementary Information:**

The online version contains supplementary material available at 10.1186/s40635-024-00610-1.

## Background

Acute Kidney Injury (AKI) is one of the most frequent complications reported in critically ill patients, and its presence substantially increases the risk of mortality [1, 2].

The relationship between lung injury and kidney function in mechanically ventilated patients is well recognized. This “lung-kidney cross talk” involves multiple factors, including direct impact of intrathoracic pressure on the right ventricle and the activation of neuro-hormonal pathways leading to retention of sodium and fluids. These mechanisms contribute to renal congestion and decreased renal perfusion, exacerbated by the vascular effects of hypercapnia and hypoxemia. Moreover, injurious mechanical ventilation can lead to biotrauma, causing renal inflammation and endothelial injury, thus resulting in further impairment in kidney function [3].

The specific effect of mechanical power, which refers to the energy load delivered to the respiratory system [4] each minute, on kidney function is poorly described. In a previous study, we demonstrated that mechanical power per se is associated with lung injury regardless of the combination of its individual components (tidal volume, respiratory rate and Positive End-Expiratory Pressure (PEEP)) [5]. However, the relative contribution of these components to the development of AKI remains unclear.

We hypothesized that individual components of mechanical ventilation, for a given mechanical power, may have distinct effects on kidney perfusion and on the risk of developing AKI. Specifically, it is likely that the PEEP component of mechanical power plays a significant role on renal congestion and mean perfusion pressure (MPP) (mean arterial pressure (MAP) minus central venous pressure (CVP)) and may be the main determinant of AKI [6–8].

The primary objective of this study was to investigate the association between the different components of mechanical power and the corresponding risk of AKI. The secondary objective was to explore the hemodynamic parameters associated with changes in mechanical power that can best predict the development of AKI.

## Materials and methods

### Study population

This was a secondary analysis of data obtained from 2 previous experimental studies focused on mechanical ventilation and ventilation-induced lung injury, involving a total of 78 pigs [5, 9].

In the first study, 36 female pigs with average weight of 23.3(± 2.3)Kg were ventilated for 48 h with an applied mechanical power ranging between 18 and 120 J/min. The tidal volume applied was equal to the functional residual capacity (strain = 1); the respiratory rate was 30 bpm, and the PEEP varied between 0 and 18cmH_2_O (0, 4, 7, 11, 14, and 18) [9].

In the second study, 42 female pigs with average weight of 24.2(± 2.0)Kg were randomized into six groups, of which three receiving low mechanical power (15 J/min) and the other three higher mechanical power (30 J/min), and then were ventilated for 48 h. In the six groups, mechanical power was delivered with different combinations of respiratory rate, tidal volume and PEEP. The applied tidal volume ranged between 0.5 and 3.8L, the respiratory rate from 5 to 44 bpm, and PEEP from 5 to 25cmH_2_O [5]. The experimental trials were previously approved by the local ethics committee (Niedersächsisches Landesamt für Verbraucherschutz und Lebensmittelsicherheit: LAVES; Oldenburg, Niedersachsen, Germany. Approval of study 1: 09/01/17; number: 16/2223; title: “Animal experimental study of the relationship between mechanical ventilation energy in the lungs and lung size”. Approval of study 2: 24/05/18; number: 18/2795; title: “Experimental confirmation of mechanical energy threshold in ventilator-induced lung injury”) and were performed in accordance with the 1975 Helsinki Declaration; the manuscript conformed to the ARRIVE guidelines [10].

Management of the study individuals and time-course of the experimental trials:

In both experiments, propofol, midazolam and sufentanil were used for the induction of anesthesia. After endotracheal intubation, standardized baseline mechanical ventilation settings were applied (Vt 6 mL/kg, PEEP 5cmH_2_O, respiratory rate to maintain PaCO_2_ between 35 and 45 mmHg). Animals were instrumented with the following devices: orogastric probe with esophageal pressure monitoring system (Nutrivent, Sidam Srl., Modena, Italy); central venous catheter; Swan-Ganz catheter; central arterial PiCCO® (Pulsion Medical System, Germany) catheter and urinary catheter.

Once the animal was clinically stable and ready for the experiment, baseline measurements were obtained and, subsequently, the settings of mechanical ventilation was modified according to the experimental group allocation and maintained unchanged throughout the experiment. In both trials, measurements were collected at 0.5 h and, subsequently, every 6 h until the end of the experiment. Plasma and urine samples were collected at baseline, 6, 12, 24, and 48 h.

The infusion of a balanced crystalloid solution (Sterofundin®; Braun GmbH, Germany) was initiated in all animals before the baseline measurement, at rate 2 mL/h. Whenever clinical signs of hypovolemia/hypoperfusion were detected (MAP < 60 mmHg or increased arterial lactates), additional boluses of 250 mL of crystalloids were delivered. In the absence of fluid responsiveness, a continuous infusion of norepinephrine or epinephrine was initiated to maintain hemodynamic stability.

At the end of the experiment, the animal was euthanized, an autopsy was performed, and three tissues samples were harvested from each lung (basal, medial, apical), together with a tissue sample from the liver, kidneys, bowel and muscle. Each specimen (approximately 2 g) was weighted before and after being heated and dried in an oven at 50 °C for 24 h to obtain the wet-to-dry ratio.

### Measured and derived variables

The following variables were calculated at each time-point of the study:

- Respiratory system mechanical power (MP_RS_) [4]:

1 $${{\text{MP}}}_{{\text{RS}}} \left({\text{J}}/{\text{min}}\right)=0.098*{\text{RR}}*\left({{\text{Vt}}}^{2}*\left(0.5*{{\text{E}}}_{{\text{RS}}}+{\text{RR}}* \frac{1 +\mathrm{ I}:{\text{E}}}{60 *\mathrm{ I}:{\text{E}}}*{{\text{R}}}_{{\text{aw}}}\right)+{\text{Vt}}*{\text{PEEP}}\right)$$

RR: respiratory rate; Vt: tidal volume; E_RS_: respiratory system elastance; I:E: inspiratory/expiratory ratio; R_aw_: airway resistances; PEEP: positive end-expiratory pressure.

- Relative components of mechanical power: the classic equation of mechanical power was partitioned to compute the amount of each of the three components of mechanical power:

*Elastic component of mechanical power* [4]:

2 $${{\text{MP}}}_{{\text{RS}}-\mathrm{Elastic component}} \left({\text{J}}/{\text{min}}\right)=0.098*{\text{RR}}*{{\text{Vt}}}^{2}*0.5*{{\text{E}}}_{{\text{RS}}}$$

*Resistive component of mechanical power* [4]:

3 $${{\text{MP}}}_{{\text{RS}}-\mathrm{Resistive component}} ({\text{J}}/{\text{min}})=0.098*{{\text{RR}}}^{2}*{{\text{Vt}}}^{2}*\frac{1 +\mathrm{ I}:{\text{E}}}{60 *\mathrm{ I}:{\text{E}}}*{{\text{R}}}_{{\text{aw}}}$$

*PEEP component of mechanical power* [4]:

4 $${{\text{MP}}}_{{\text{RS}}-\mathrm{PEEP component}} \left({\text{J}}/{\text{min}}\right)=0.098*{\text{RR}}*{\text{Vt}}*{\text{PEEP}}$$

Mean perfusion pressure (MPP):

5 $$\mathrm{MPP }({\text{mmHg}})=\mathrm{ MAP}-{\text{CVP}}$$

*MAP* mean arterial pressure, *CVP* central venous pressure;

### Outcome variables

The animals were grouped into four categories according to the value of plasma creatinine assessed at the end of the experiment, based on the RIFLE criteria for kidney injury [11]. The experimental groups are as follows: 1) No AKI: no increase in creatinine compared to baseline; 2) RIFLE 1-Risk: increase of creatinine of > 50%; 3) RIFLE 2-Injury: a two-fold increase in creatinine; 4) RIFLE 3-Failure: a three-fold increase in creatinine.

### Statistical analysis

Data are reported as median (± 95% confidence interval). Continuous variables were compared using one-way ANOVA. The time-course description of the variables was evaluated via a linear mixed-effects model, with the severity of AKI, time, and their interaction as fixed effects, and the single individual as random effect. To reflect the weight of each variable according to the time of exposure, the time-weighted average of each study period was computed as the area under the curve of the variable.

The association between renal function and the hemodynamic variables was explored with a regression model, where the time-weighted average hemodynamic value recorded during the experimental phase was plotted against the increase in serum creatinine. The strength of the association was also evaluated with a Receiver Operating Characteristic model.

Two-tailed *p*-value < 0.05 was considered statistically significant. All analyses were performed with SPSS 25 (SPSS inc., Chicago, IL).

## Results

**Fig. 1:**
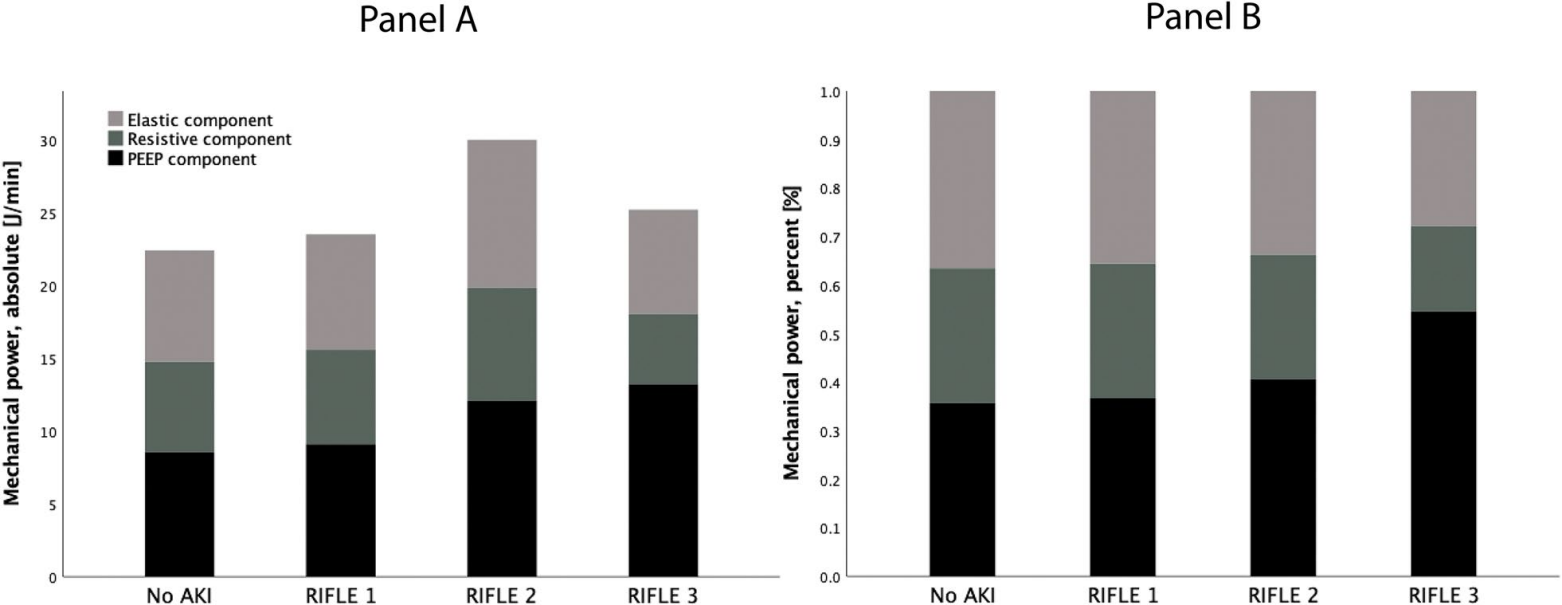
Applied mechanical power throughout the experiment (**A** absolute values; **B** percentage values), detailed according to the elastic, resistive and PEEP components of mechanical power, and grouped by the severity of renal impairment. As shown, the PEEP component of the mechanical power showed the strongest association with renal failure: *Absolute values:* Elastic: NO AKI 7.6 (3.1), RIFLE 1-Risk 7.9 (3.4), RIFLE 2-Injury 10.2 (4.5), RIFLE 3-Failure 7.2 (3.5); *p* = 0.072; Resistive: 6.2 (3.1), 6.5 (3.5), 7.7 (3.9) 4.8 (3.6) *p* = 0.130; PEEP: 8.6 (6.0), 9.1 (6.3), 12.1 (6.2), 13.2 (5.4) *p* = 0.052. *Percentage values:* Elastic: 37 (11), 36 (12), 34 (10), 28 (8). *p* = 0.078; Resistive: 28 (8), 28 (10), 26 (11) 18 (11) *p* = 0.010; PEEP: 36 (15), 37 (16), 41 (16), 55 (16) *p* = 0.003)

The mechanical power was the independent variable; therefore, in Fig. 1, we present the association between kidney function and applied mechanical power, detailed as its three components and expressed as absolute (Panel A) and relative values (Panel B). The total mechanical power ranged between 22.4(± 10.7) and 29.9(± 10.1)J/min. The mechanical power component due to the applied PEEP showed the strongest association with AKI, either when assessed as absolute (*p* = 0.052) or as a proportion of the total power (*p* = 0.003). Kidney function worsened from NO AKI to RIFLE 3-Failure with an increase in MP due to an increase in PEEP. In the logistic regression, only PEEP was significantly associated with AKI, while respiratory rate and tidal volume, the two other primary components of mechanical power, were not (see Additional file 1: Table S1 in the supplementum).

**Fig. 2:**
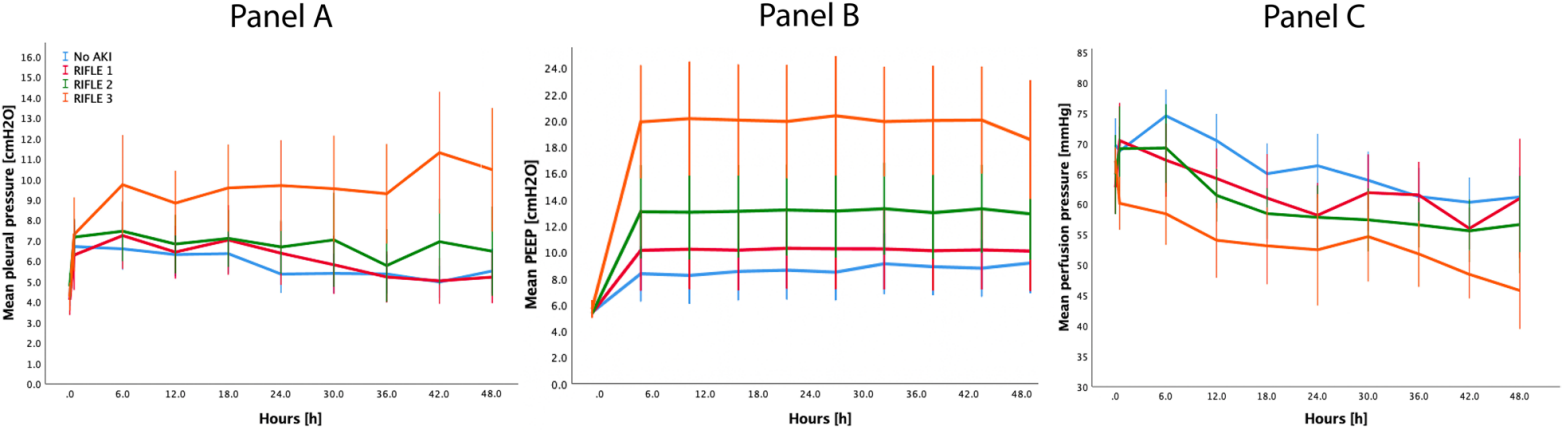
Time-course of mean pleural pressure (**A** difference between groups, *p* = 0.094; difference between time-points, *p* < 0.001; interaction between groups and time-points, *p* < 0.001)), PEEP (**B** difference between groups, *p* < 0.001; difference between time-points, *p* < 0.001; interaction between groups and time-points, *p* < 0.001) and mean perfusion pressure (**C** difference between groups, *p* < 0.001; difference between time-points, *p* < 0.001; interaction between groups and time, *p* < 0.001), throughout the experiment, according to the severity of acute kidney injury

The time-course of mean pleural pressure, the possible link variable mediating the effects of mechanical power on AKI, is presented in Fig. 2, Panel A. As shown, mean pleural pressure was higher in group RIFLE 3, although such difference did not reach the statistical significance (*p* = 0.094). However, the changes in mean pleural pressure were significantly different over time (*p* < 0.001), and a significant interaction was found between time and AKI groups (*p* < 0.001). In the RIFLE 3 group, pleural pressure progressively increased over time, while it remained stable or even decreased in the remaining groups.

Determinants of acute kidney injury:

Table 1 reports the most relevant anatomical and physiological study variables, grouped according to the different stages of AKI and collected at baseline before applying the experimental mechanical power. As shown, in pigs of similar body weight (*p* = 0.142) and lung volume (*p* = 0.436), ventilated at similar mechanical power of approximately 6J/min (*p* = 0.393), the respiratory, hemodynamic and gas-exchange variables were also similar, but a 20 mL difference in tidal volume in the RIFLE 3 group (*p* = 0.038).

**Table 1:** Anatomical and physiological study variables collected at baseline, according to RIFLE classification

Variable	NO AKI (*n*: 27)	RIFLE 1: risk (*n*: 18)	RIFLE 2: injury (*n*: 18)	RIFLE 3: failure (*n*: 15)	*p*-value
Baseline characteristics					
Initial weight (Kg)	24.4 (± 2.3)	23.2 (± 2.2)	23.1 (± 1.7)	24.2 (± 2.2)	0.142
Functional residual capacity (mL)	352 (± 64)	348 (± 66)	363 (± 61)	380 (± 49)	0.436
Respiratory variables					
Tidal volume (mL)	243 (± 25)	230 (± 27)	241 (± 29)	220 (± 25)	0.038
Respiratory rate (bpm)	21 (± 4)	21 (± 2)	20 (± 3)	21 (± 3)	0.894
Mean airway pressure (cmH_2_O)	9.8 (± 2.1)	9.1 (± 0.9)	9.5 (± 1.7)	9.3 (± 1.2)	0.467
PEEP measured (cmH_2_O)	5.4 (± 0.7)	5.4 (± 0.3)	5.3 (± 0.3)	5.7 (± 1.3)	0.541
Mean pleural pressure (cmH_2_O)	4.3 (± 1.3)	4.1 (± 1.6)	4.5 (± 1.7)	4.3 (± 1.3)	0.866
Driving pressure (cmH_2_O)	3.3 (± 1.4)	3.0 (± 1.0)	3.1 (± 0.8)	2.7 (± 0.8)	0.538
Stress-Transpulmonary pressure (mmHg)	5.15 (± 1.61)	4.57 (± 1.62)	4.82 (± 1.84)	4.3 (± 2.29)	0.513
Strain, fraction	0.71 (± 0.14)	0.68 (± 0.11)	0.68 (± 0.15)	0.59 (± 0.10)	0.045
Airway resistance (cmH_2_O*min/L)	0.18 (± 0.05)	0.20 (± 0.08)	0.18 (± 0.05)	0.17 (± 0.03)	0.405
Elastance, respiratory system (cmH_2_O/mL)	39.0 (± 6)	38.4 (± 6.0)	39.6 (± 7.9)	40.0 (± 9.8)	0.926
Elastance, lung (cmH_2_O/mL)	21.80 (± 6.65)	21.36 (± 8.17)	20.08 (± 6.23)	21.75 (± 8.45)	0.877
Elastance, chest-wall (cmH_2_O/mL)	17.10 (± 4.96)	17.07 (± 6.43)	19.48 (± 5.01)	18.30 (± 7.39)	0.530
Specific elastance	7.28 (± 1.90)	6.82 (± 2.30)	7.05 (± 1.65)	7.39 (± 3.42)	0.892
Mechanical power, respiratory system (J/min)	6.7 (± 2.0)	6.0 (± 1.3)	6.3 (± 1.6)	5.9 (± 1.4)	0.393
Mechanical power, lung (J/min)	4.42 (± 1.77)	3.94 (± 1.21)	3.99 (± 1.28)	3.77 (± 1.11)	0.480
Hemodynamic					
Heart rate (bpm)	98 (± 19)	98 (± 21)	100 (± 13)	95 (± 20)	0.964
Mean arterial pressure (mmHg)	77 (± 9)	70 (± 8)	73 (± 4)	73 (± 5)	0.070
Central venous pressure (mmHg)	8 (± 3)	7 (± 3)	8 (± 5)	6 (± 1)	0.470
Mean perfusion pressure (mmHg)	70 (± 9)	63 (± 6)	65 (± 9)	67 (± 4)	0.150
Mean pulmonary artery pressure (mmHg)	21 (± 4)	18 (± 2)	21 (± 4)	19 (± 3)	0.254
Cardiac output (L/min)	3.98 (± 0.72)	3.81 (± 0.91)	4.6 (± 0.66)	4.07 (± 0.62)	0.219
Central venous oxygen saturation (%)	84 (± 5)	85 (± 4)	89 (± 3)	87 (± 3)	0.065
Lactates (mg/dL)	0.57 (± 0.20)	0.57 (± 0.21)	0.68 (± 0.63)	1.29 (± 2.69)	0.592
Blood gas analysis					
PaO2 (mmHg)	227 (± 11)	231 (± 23)	232 (± 15)	227 (± 16)	0.840
PaCO2 (mmHg)	44 (± 5)	44 (± 3)	44 (± 4)	43 (± 4)	0.977
pH	7.51 (± 0.05)	7.52 (± 0.03)	7.47 (± 0.06)	7.52 (± 0.04)	0.274

*p*-value computed by one-way ANOVA

In Table 2, we present the same variables collected 30 min after the application of the experimental high mechanical power ventilation. In the animal groups which developed different degrees of AKI severity, from NO AKI to RIFLE3-failure, several respiratory mechanical variables were remarkably different across groups. Indeed, respiratory system (*p* = 0.011) and lung (*p* = 0.010) mechanical power, driving pressure (*p* = 0.027), mean airway pressure (*p* < 0.001), PEEP (*p* < 0.001), lung stress (*p* = 0.004), respiratory system (*p* = 0.005) and lung elastances (*p* < 0.001), as well as the specific lung elastance (*p* = 0.001), were worse in the groups with worse kidney function at end of experiment. At 0.5 h, as shown in Table 2, the only differences observed within the groups were a significantly increased heart rate and mean pulmonary pressure and lower central venous oxygen saturation, in the groups with the most severe AKI. Of note, the cardiac output was similar across groups, likely consequent to a significantly increased infusion of cardioactive drugs.

**Table 2:** Anatomical and physiological study variables collected at 0.5h, according to RIFLE classification

Variable	NO AKI (*n*: 27)	RIFLE 1-Risk (*n*: 18)	RIFLE 2-Injury (*n*: 18)	RIFLE 3-Failure (*n*: 15)	*p*-value
Respiratory variables					
Tidal volume (mL)	401 (± 215)	422 (± 189)	441 (± 201)	397 (± 197)	0.903
Respiratory rate (bpm)	29 (± 11)	26 (± 11)	26 (± 9)	21 (± 9)	0.084
Mean airway pressure (cmH_2_O)	14.2 (± 6.2)	16.2 (± 6.9)	20.7 (± 9.7)	26.2 (± 10.1)	< 0.001
PEEP measured (cmH_2_O)	8.4 (± 5.5)	10.1 (± 6.5)	13.1 (± 7.5)	19.9 (± 8.3)	< 0.001
Driving pressure (cmH_2_O)	5.5 (± 2.2)	6.5 (± 2.25)	7.7 (± 3.8)	5.4 (± 2.0)	0.027
Mean pleural pressure (cmH_2_O)	6.7 (± 2.7)	6.3 (± 3.6)	7.2 (± 1.9)	7.3 (± 3.4)	0.742
Stress-Transpulmonary pressure (mmHg)	7.8 (± 4.2)	10.6 (± 5.2)	13.6 (± 7.9)	13.8 (± 6.7)	0.004
Strain, fraction	1.13 (± 0.54)	1.21 (± 0.49)	1.20 (± 0.47)	1.02 (± 0.37)	0.687
Airway resistance (cmH_2_O*min/L)	0.17 (± 0.07)	0.19 (± 0.07)	0.20 (± 0.07)	0.23 (± 0.08)	0.060
Elastance, respiratory system (cmH_2_O/mL)	39.7 (± 11.9)	43.0 (± 12.6)	54.0 (± 28.3)	60.6 (± 25.1)	0.005
Elastance, lung (cmH_2_O/mL)	20.9 (± 10.2)	26.1 (± 8.9)	35.6 (± 27.2)	45.3 (± 21.5)	< 0.001
Elastance, chest-wall (cmH_2_O/mL)	18.8 (± 5.7)	17.0 (± 7.5)	18.5 (± 4.4)	15.3 (± 10.5)	0.435
Specific elastance	7.2 (± 3.5)	8.9 (± 3.8)	12.3 (± 8.8)	15.1 (± 7.9)	0.001
Mechanical power, respiratory system (J/min)	20.3 (± 9.4)	22.8 (± 11.4)	30.5 (± 10.7)	25.0 (± 7.8)	0.011
Mechanical power, lung (J/min)	13.1 (± 6.7)	16.3 (± 7.8)	22.1 (± 8.5)	22.4 (± 17.7)	0.010
Hemodynamic					
Heart rate (bpm)	107 (± 24)	122 (± 21)	135 (± 27)	142 (± 30)	< 0.001
Mean arterial pressure (mmHg)	78 (± 10)	81 (± 14)	80 (± 14)	71 (± 7)	0.082
Central venous pressure (mmHg)	10 (± 4)	10 (± 5)	11 (± 5)	11 (± 3)	0.613
Mean perfusion pressure (mmHg)	69 (± 10)	70 (± 13)	69 (± 15)	60 (± 8)	0.066
Mean pulmonary artery pressure (mmHg)	23 (± 5)	24 (± 9)	27 (± 8)	31 (± 7)	0.016
Cardiac output (L/min)	4.24 (± 1.17)	4.14 (± 0.83)	4.03 (± 1.44)	4.25 (± 1.24)	0.943
Central venous oxygen saturation (%)	83 (± 7)	88 (± 6)	83 (± 8)	79 (± 10)	0.028
Lactates (mg/dL)	1.00 (± 0.54)	1.11 (± 0.51)	1.17 (± 0.91)	1.40 (± 1.12)	0.440
Infused catecholamines (mcg/Kg)	0.013 (± 0.029)	0.023 (± 0.045)	0.072 (± 0.114)	0.075 (± 0.056)	0.005
Blood gas analysis					
PaO2 (mmHg)	232 (± 26)	248 (± 18)	246 (± 20)	238 (± 22)	0.336
PaCO2 (mmHg)	27 (± 9)	26 (± 9)	26 (± 12)	32 (± 14)	0.093
pH	7.65 (± 0.14)	7.70 (± 0.10)	7.69 (± 0.13)	7.60 (± 0.16)	0.143

*p*-value computed by one-way ANOVA

Table 3 reports the same set of previously assessed variables, collected after 48 h of high mechanical power mechanical ventilation. As shown, most of the mechanical variables which differed at the beginning of the experiment, became similar across groups at 48 h, due to progressive worsening in AKI groups 1 and 2. Indeed, only PEEP (*p* = 0.001) and mean airway pressure (*p* = 0.001) remained different. Of note, however, the pleural pressure significantly deteriorated in the RIFLE 3 group. At 48 h, cardiac output (*p* < 0.001) and CVP (*p* = 0.013) increased with worse AKI, while the MPP significantly decreased (*p* = 0.036). The time-course of MAP, MPP, CVP and cardiac output is reported in Additional file 1: Fig. S1, while Additional file 1: Fig. S2 depicts the evolution of fluid infusion, urine production, fluid balance and creatinine, throughout the experiment.

**Table 3:** Anatomical and physiological study variables collected at 48h, according to RIFLE classification

Variable	NO AKI (*n*: 25)	RIFLE 1: risk (*n*: 15)	RIFLE 2: injury (*n*: 14)	RIFLE 3: failure (*n*: 10)	*p*-value
Respiratory variables					
Tidal volume (mL)	413 (± 219)	398 (± 175)	434 (± 218)	372 (± 134)	0.893
Respiratory rate (bpm)	29 (± 12)	26 (± 11)	26 (± 9)	21 (± 9)	0.084
Mean airway pressure (cmH_2_O)	15.8 (± 6.2)	15.9 (± 5.7)	20.4 (± 8.3)	26.2 (± 8.3)	0.001
PEEP measured (cmH_2_O)	9.2 (± 6.0)	10.1 (± 6.4)	12.9 (± 7.4)	18.5 (± 8.7)	0.001
Driving pressure (cmH_2_O)	7.2 (± 3.6)	7.8 (± 2.8)	7.9 (± 2.6)	5.6 (± 2.6)	0.120
Mean pleural pressure (cmH_2_O)	5.2 (± 2.7)	5.2 (± 2.5)	6.0 (± 4.2)	10.5 (± 3.8)	< 0.001
Stress-Transpulmonary pressure (mmHg)	11.8 (± 5.2)	12.4 (± 4.8)	14.7 (± 8.8)	11.5 (± 3.8)	0.472
Strain, fraction	1.45 (± 0.89)	1.15 (± 0.55)	1.70 (± 1.17)	1.26 (± 0.50)	0.348
Airway resistance (cmH_2_O*min/L)	0.20 (± 0.09)	0.22 (± 0.08)	0.21 (± 0.05)	0.23 (± 0.09)	0.740
Elastance, respiratory system (cmH_2_O/mL)	48.9 (± 18.0)	52.1 (± 19.6)	49.6 (± 12.6)	56.1 (± 14.8)	0.693
Elastance, lung (cmH_2_O/mL)	31.8 (± 16.3)	36.9 (± 19.4)	34.2 (± 7.9)	34.1 (± 13.4)	0.872
Elastance, chest-wall (cmH_2_O/mL)	15.8 (± 6.0)	16.2 (± 5.8)	15.3 (± 7.1)	22.0 (± 8.4)	0.069
Specific elastance	9.23 (± 4.57)	12.69 (± 6.97)	9.69 (± 3.03)	9.94 (± 4.21)	0.192
Mechanical power, respiratory system (J/min)	24.7 (± 13.0)	23.7 (± 7.9)	29.9 (± 12.5)	25.4 (± 8.8)	0.461
Mechanical power, lung (J/min)	18.9 (± 10.8)	18.3 (± 7.5)	23.3 (± 9.7)	17.6 (± 7.2)	0.401
Hemodynamic					
Heart rate (bpm)	86 (± 21)	87 (± 21)	109 (± 23)	115 (± 20)	< 0.001
Mean arterial pressure (mmHg)	72 (± 12)	71 (± 19)	71 (± 14)	61 (± 9)	0.186
Central venous pressure (mmHg)	10.7 (± 3.8)	10.3 (± 4.7)	14.2 (± 5.6)	15.1 (± 4.9)	0.013
Mean perfusion pressure (mmHg)	61 (± 12)	61 (± 19)	57 (± 15)	46 (± 10)	0.036
Mean pulmonary artery pressure (mmHg)	25 (± 7)	24 (± 8)	32 (± 8)	34 (± 6)	0.001
Cardiac output (L/min)	2.86 (± 0.77)	2.60 (± 0.65)	4.11 (± 1.74)	4.57 (± 1.86)	< 0.001
Central venous oxygen saturation (%)	77 (± 9)	76 (± 8)	73 (± 16)	81 (± 5)	0.394
Lactates (mg/dL)	0.63 (± 0.29)	0.62 (± 0.259)	1.42 (± 1.62)	1.39 (± 0.79)	0.011
Infused catecholamines (mcg/Kg)	4.3 (± 6.0)	4.4 (± 5.7)	12.9 (± 15.3)	11.0 (± 17.0)	0.034
Blood gas analysis					
PaO2 (mmHg)	222 (± 28)	229 (± 189)	213 (± 46)	227 (± 27)	0.548
PaCO2 (mmHg)	21 (± 10)	18 (± 4)	24 (± 20)	30 (± 20)	0.259
pH	7.57 (± 0.06)	7.59 (± 0.05)	7.52 (± 0.14)	7.45 (± 0.16)	0.005

Because 14 study-individuals expired before hour 48, the analysis was performed on 64 animals; p-value computed by one-way ANOVA

In Table 4, the fluid and sodium balances in the four AKI groups are displayed, as well as the lung weight and the wet-to-dry ratio of lungs, kidney, liver, bowel and muscles. As shown, the fluid balance was significantly different between groups (*p* = 0.002) while no difference was observed in lung weight (*p* = 0.080). The wet-to-dry ratio of kidneys (*p* < 0.001) and liver (*p* = 0.041) were significantly different in the four groups; conversely, lung, bowel and muscle wet-to-dry ratio were similar (respectively, *p* = 0.622; *p* = 0.983 and *p* = 0.551).

**Table 4:** Post-mortem analysis, indexed lungs weight and wet-to-dry ratios, according to RIFLE classification

Variable	NO AKI (*n*: 27)	RIFLE 1: risk (*n*: 18)	RIFLE 2: injury (*n*: 18)	RIFLE 3: failure (*n*: 15)	*p*-value
Fluid balance (mL)	3.48 (± 2.56)	2.74 (± 1.75)	4.04 (± 3.65)	6.89 (± 4.52)	0.002
Sodium retention (mEq)	658 (± 348)	526 (± 239)	785 (± 539)	1435 (± 479)	< 0.001
Lungs’ weight (g/Kg_BW_)	21.6 (7.0)	18.4 (4.7)	20.5 (8.0)	24.5 (6.7)	0.080
Wet-to-dry lungs	6.48 (0.71)	6.72 (1.19)	6.56 (0.97)	6.86 (1.06)	0.622
Wet-to-dry kidneys	4.70 (1.02)	5.40 (0.83)	5.30 (0.66)	6.01 (0.47)	< 0.001
Wet-to-dry liver	3.88 (0.40)	4.31 (0.52)	4.04 (0.45)	4.20 (0.45)	0.041
Wet-to-dry bowel	5.65 (0.84)	5.52 (0.49)	5.61 (0.69)	5.59 (1.44)	0.983
Wet-to-dry muscle	3.78 (0.72)	3.92 (0.53)	4.00 (0.64)	3.68 (0.58)	0.551

*p*-value computed by one-way ANOVA

Arterial, central venous and mean perfusion pressure, and acute kidney injury:

The association between plasma creatinine measured at 48 h and MAP, MPP and CVP was explored with a linear regression model (Additional file 1: Fig. S3): MPP showed greater association (*p* < 0.001; *R*^2^ 0.265) compared to MAP (*p* < 0.001; *R*^2^ 0.162) and CVP (*p* = 0.004; *R*^2^ 0.106). The same association was also tested by Receiver Operating Characteristic model (Additional file 1: Fig. S4), where the outcome was presence or absence of kidney injury (absence defined as pertaining to groups: NO AKI and RIFLE 1-Risk; presence defined as pertaining to groups RIFLE 2-Injury and RIFLE 3-Failure). MPP showed the highest association (AUC[95%CI] 0.765[0.657–0.873]), compared to MAP (AUC[95%CI] 0.690[0.567–0.814]) and CVP (AUC[95%CI] 0.711[0.591–0.831]).

Likewise, in a linear regression model, the strongest association with the kidneys’ wet-to-dry ratio was observed for MPP (*p* = 0.002; *R*^2^ 0.145), followed by CVP (*p* = 0.023; *R*^2^ 0.088) and MAP (*p* = 0.049; *R*^2^ 0.062) (Additional file 1: Fig. S5).

## Discussion

The main findings of the present analysis are as follows: 1) similar mechanical power, regardless its components, produced similar lung injury (lung weight and wet-to-dry ratio). In contrast, for a similar mechanical power, the prevalence of the PEEP component led to different severity of acute kidney injury (creatinine and wet-to-dry of the kidneys); 2) a reduced MPP is a better renal failure predictor than its components (MAP and CVP) taken separately.

### Determinants of acute kidney injury

As shown in Table 2, immediately after the experiment initiation, mechanical power was significantly different between groups. Notably, however, the RIFLE 3-Failure group received a lower amount of mechanical power compared with the RIFLE 2-Injury group (25.0 (± 7.8)J/min vs. 30.5(± 10.7)J/min, respectively), suggesting that mechanical power, per se, is not the main determinant of kidney impairment. Consequently, we partitioned the total mechanical power into its three determinants[4]: elastic (determined by the tidal volume), resistive (determined by the resistance of the airway) and PEEP component. The analysis of the single components strongly suggested that the PEEP component is the primary determinant of acute kidney injury, compared to the tidal volume and the respiratory rate.

The intermediate variable linking mechanical power, PEEP, and acute kidney failure is likely the increased mean pleural pressure, which, in turn, alters the mean perfusion pressure by decreasing MAP and increasing CVP (see supplementum for the relationship between PEEP and MPP (Additional file 1: Fig. S6) and PEEP and pleural pressure (Additional file 1: Fig. S7)). Interestingly, at 0.5h, MAP, CVP and cardiac output were similar in the four groups, as well as the mean pleural pressure. This suggests an early adaptation in the hemodynamic response to the stressful condition imposed by the mechanical power. However, at the end of the experiment, the overall respiratory and hemodynamic panels were different. Indeed, while the early response to the increased mechanical power was primarily an impairment of lung mechanics with near-normal hemodynamic, at 48h the lung mechanics were similarly impaired across the four groups. This suggests similar lung damage. In contrast, the hemodynamic variables and mean pleural pressure were markedly different, likely accounting for the different magnitude of kidney damage. Of note, despite a higher amount of fluids and catecholamines administered across groups, the overall hemodynamic pattern showed a progressive deterioration going from group NO AKI to RIFLE 3. The finding of a higher cardiac output in the RIFLE 3-Failure group likely reflects the increased use of fluids and cardioactive drugs administered to maintain hemodynamic targets.

In a previous study[5], we observed that mechanical power > 20J/min led to similar lung damage regardless its composition (i.e., a relatively low PEEP, in the range of 5–8cmH_2_O, associated with tidal volume of 30ml/Kg, showed similar lung damage compared to a setting of tidal volume of 10mL/Kg and PEEP > 20cmH_2_O). In this study, lung edema quantified by the end-experimental lung-weight and the wet-to-dry ratio was similar in the four AKI groups. Conversely, kidney damage was different across groups, and this was primarily associated to the applied PEEP. These data, considered all together, suggest that the high mechanical power induced similar pulmonary damage regardless its composition. However, when the PEEP component is predominant (around 20cmH_2_O in our study population), the systemic effects are remarkably different. Higher PEEP, indeed, was associated with significantly higher venous congestion (as assessed by CVP and previously suggested in the literature [12, 13], fluid and sodium retention and a significant increase of the kidney and liver wet-to-dry ratios. These data highlight the risk of high PEEP, as also shown by a randomized trial where higher PEEP was associated with worse outcomes[14].

Although the investigation of the possible harmful effects of PEEP is usually focused primarily on the respiratory system, our findings emphasize the detrimental extrapulmonary consequences of PEEP. Even though the hemodynamic effects of PEEP are well known since decades [15–18], these implications are usually underestimated as the impaired hemodynamics are masked by the use of fluids or cardioactive drugs. Indeed, in this study, the cardiac output was even higher in the groups with the most severe degree of kidney failure. Therefore, when advocating for a liberal versus conservative fluid management strategy, the extrapulmonary effects of the respiratory treatment should be taken into account.

It is not clear why, despite similar levels of mean airway pressure, higher PEEP is associated with more severe extrapulmonary effects compared to high tidal volume. A possible explanation may be that the impediment to venous return is constant and continuous compared with the cyclical effect of tidal volume.

### Pathophysiology of acute kidney injury in mechanical ventilation

Several studies investigated the possible predictive marker of kidney failure. Within them, the most investigated variable was MAP [19–22]. Traditionally, MAP < 65mmHg has been considered a risk factor for kidney hypoperfusion and failure [23, 24]. In septic patients, it has been suggested that MPP could be more accurate than the MAP in the prediction of the renal outcome [6–8]. The physiological basis of perfusion pressure is sound: a high CVP is associated with proportionally higher renal venous pressure, leading both to the decrease of kidney perfusion and to an increased congestion/edema of the kidney and may be a more insidious and less recognized cause of renal injury compared to MAP. Given that MPP showed the strongest association both with AKI severity and the kidney’s wet-to-dry ratio, the assessment of the arterial and venous sides of kidney’s circulation allows a more accurate prediction of AKI. Additional file 1: Fig. S8 depicts the hypothetical physiological mechanisms underlying the development of acute kidney injury, in ventilated individuals.

The implications of such finding are relevant: the dogma that “fluid infusion optimizes renal perfusion and performance” may be simply wrong. Because the gradient between arterial and venous pressures has a significant role in the development of kidney injury, a cautious use of fluids, inotropes and perhaps venous vasodilators could be the key to optimize renal performance and to prevent kidney vascular congestion.

## Limitations of the study

The most relevant limitation of the present study is its retrospective nature and the lack of a defined protocol for hemodynamic management. Another limitation is the lack of pathological assessment on the kidney specimens for the confirmation and quantification of kidney failure, and to define the type of damage that the experimental subjects incurred (e.g., glomerular vs. tubular).

## Conclusions

Within the components of harmful mechanical ventilation, as defined by the level of applied mechanical power, high PEEP is the greatest risk factor for AKI development. Within the hemodynamic predictors of kidney failure during mechanical ventilation, the combination of MAP and CVP into the MPP provides a predictive advantage, compared to MAP and CVP taken separately.

## Take-home message

PEEP was the only variable included in the mechanical power associated with kidney failure. This may be secondary to the hemodynamic effects of PEEP on central venous pressure (CVP), which results in a reduction of the mean perfusion pressure (mean arterial pressure  minus CVP).

### Supplementary Information

**Additional file 1:** The supplemental material reports further details on the methodology we used to carried out the experiment and the analysis, as well as further results.

## Data Availability

The data that support the findings of this study are available from the corresponding author, upon reasonable request.

## Abbreviations

AKI: Acute kidney injury
PEEP: Positive end-expiratory pressure
MPP: Mean perfusion pressure
MAP: Mean arterial pressure
CVP: Central venous pressure
