# Supplementary material for: Determinants of acute kidney injury during high-power mechanical ventilation: secondary analysis from experimental data
Source: Intensive Care Med Exp. 2024 Mar 21;12:31. doi: 10.1186/s40635-024-00610-1 (PMC10957825; doi:10.1186/s40635-024-00610-1)
Supplement: Supplementary file 1 — Additional file 1: The supplemental material reports further details on the methodology we used to carried out the experiment and the analysis, as well as further results. [file 40635_2024_610_MOESM1_ESM.docx]

**Determinants of acute kidney injury during high-power mechanical ventilation: secondary analysis from experimental data**

Simone Gattarello^1^ MD, PhD; Fabio Lombardo^1^ MD; Federica Romitti^2^ MD; Rosanna D’Albo^2^ MD; Mara Velati^1^ MD; Isabella Fratti^2^ MD; Tommaso Pozzi^2^ MD; Rosmery Nicolardi^1^ MD; Antonio Fioccola^2^ MD; Mattia Busana^2^ MD; Francesca Collino^3^ MD; Peter Herrmann^2^ M.Sc.; Luigi Camporota^4^ MD; Michael Quintel^2,5^ MD; Onnen Moerer^2^ MD; Leif Saager^2^ MD; Konrad Meissner^2^ MD; and Luciano Gattinoni^2^ MD, FRCP;

Additional file 1

**Corresponding author:**

Dr. Simone Gattarello. Department of Anesthesia and Intensive Care Medicine, IRCCS San Raffaele Scientific Institute. Via Olgettina 60; 20132; Milan; Italy.

Phone: +39 02 26432 656; E-mail: [gattarello@gmail.com](mailto:gattarello@gmail.com)

**Material and methods**

*Measured variables:*

The following variables were collected throughout the study. Respiratory: setting of mechanical ventilation and the derived intrathoracic pressure (mean airway pressure, peak, inspiratory plateau and expiratory plateau pressures, driving pressure). Hemodynamic: heart rate, arterial and pulmonary pressures, central venous pressure, central venous oxygen saturation, infused catecholamines. Blood gas-analysis of arterial, venous and mixed samples. Laboratory: markers of renal failure were collected at baseline, 6, 12, 24 and 48 hours.

Wet-to-dry ratio was calculated as follow: each sample (approx. 2 g of weight) of lungs, kidney, bowel, liver and muscle was weighted before and after being heated and dried in an oven at 50 degrees, during 24 hours.

*Other derived variables:*

- Static respiratory system elastance (E_RS_):

$E_{RS} ({cmH}_{2}O/mL)= \frac{P_{driv}}{Vt}$ (1)

P_driv_: driving pressure; Vt tidal volume.

- Mean pleural pressure (P_pl.mean_):

$P_{pl.mean} {(cmH}_{2}O)= \frac{E_{cw}}{E_{rs}}*P_{aw.mean}$ (2)

E_cw_: chest-wall elastance; E_rs_: respiratory system elastgance; Paw.mean: mean airway pressure.

- Airway resistance (R_AW_):

$R_{AW}({cmH}_{2}O*min/L)= \frac{P_{peak} -P_{plat}}{Flow}$ (3)

P_peak_: peak pressure; P_plat_: plateau pressure.

- Fluid balance: assessed at each measurement as the total amount of infused fluids (hydration maintenance, infused drugs and all fluid challenges that were delivered) minus the urinary output and blood samples.

- Sodium balance (Na^+^_ret_): assessed at 0, 6, 12, 24 and 48 hours; computed as:

${\mathrm{Na}^{+}}_{\mathrm{ret}} \left( \mathrm{mmol} \right)={{[Na}^{+}]}_{\inf}*V_{\inf}- {{[Na}^{+}]}_{u}*V_{u}$ (4)

[Na^+^]_inf_: plasmatic sodium concentration; V_inf_: amount of infused solutions; [Na^+^]_u_: urine sodium concentration; V_u_: volume of urine.

- Functional residual capacity (FRC): calculated by the mean of a simplified helium-dilution technique, according to the following equation:

$FRC = V_{\mathrm{start}}\frac{C_{\mathrm{start}}- C_{\mathrm{end}}}{C_{\mathrm{end}}}$ (5)

V_start_: volume of helium gas-mixture volume that was insufflated within the animals’ lungs; C_start_: initial concentration of helium within the balloon before the insufflation; C_end_: helium concentration in the exhaled air, after 1 minute of rebreathing.

*Esophageal pressure measurement:*

Esophageal pressure was measured with a radio-opaque catheter equipped with a balloon in the lower part (Nutrivent, Sidam Srl., Modena, Italy), that was filled with 4 mL of air, as recommended by the manufacturer. The catheter was introduced trans-orally and advanced to reach the stomach (position confirmed by: 1. suction of gastric secretions and 2. rise in intra-abdominal pressure following external manual epigastric compres­sion). Then, it was retracted into the esophagus (i.e., confirmed by the presence of cardiac arti­facts in the pressure tracing and by the difference in the absolute pressure), in the lower third of the esophagus. All traces were processed and displayed on a dedicated data acquisition system (Optivent SIDAM Srl, Modena, Italy). Here below we report an example of esophageal pressure tracing:

**
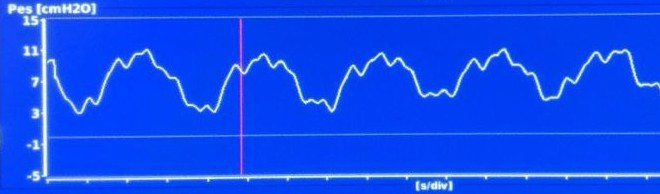
**

The recording of the tidal esophageal pressure was performed as follows: observation of tidal volume during at least 1 minute; whether the respiratory pattern was stable and the morphology of the esophageal pressure wave was unchanged over following breaths, the value was recoded. The only criteria to exclude a breath was the presence of abnormal morphology of the esophageal pressure curve. In this situation, the investigator waited the animal to have a stable breathing pattern and collected such value.

The correct positioning and calibration of the esophageal probe was confirmed by the Baydur Maneuver, as previously reported (Baydur et al. A simple method for assessing the validity of the esophageal balloon technique. Am Rev Respir Dis. 1982. 10.1164/arrd.1982.126.5.788).

**Supplemental figures**

**Figure S1:** Time-course of mean arterial pressure (Panel A: difference between groups, p<0.001; difference between time-points, p<0.001; interaction between groups and time-points, p=0.691), mean perfusion pressure (Panel B: difference between groups, p<0.001; difference between time-points, p<0.001; interaction between groups and time, p=0.662), central venous pressure (Panel C: difference between groups, p=0.047; difference between time-points, p=0.012; interaction between groups and time, p<0.001) and cardiac output (Panel D: difference between groups, p=0.757; difference between time-points, p<0.001; interaction between groups and time, p<0.001), throughout the experiment.


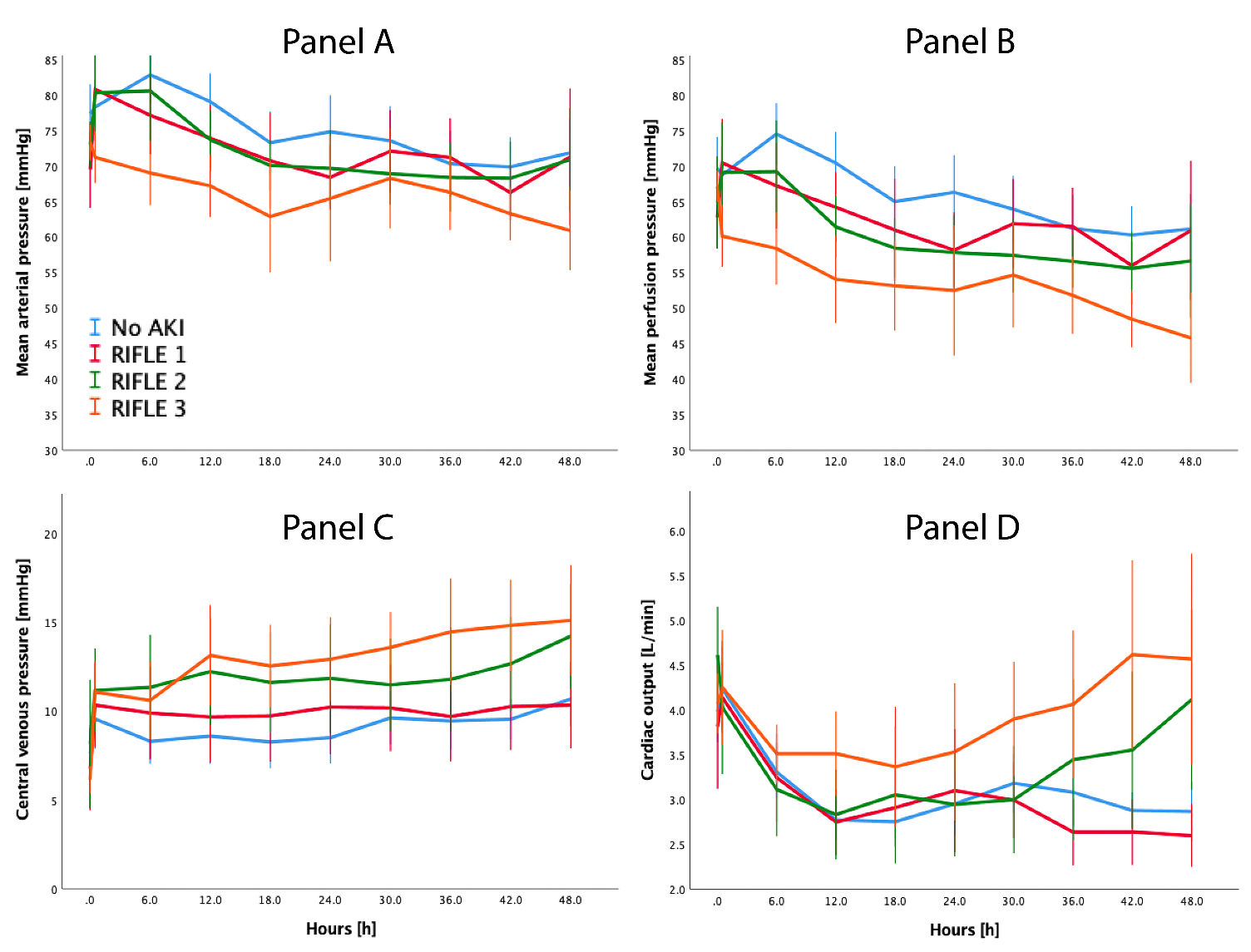


**Figure S2:** Time-course of infused fluids, (Panel A: difference between groups, p=0.049; difference between time-points, p<0.001; interaction between groups and time-points, p=0.073), urine production (Panel B: difference between groups, p=0.888; difference between time-points, p<0.001; interaction between groups and time, p<0.001), cumulative fluid balance (Panel C: difference between groups, p=0.076; difference between time-points, p<0.001; interaction between groups and time, p<0.001) and plasma creatinine (Panel D: difference between groups, p=0.044; difference between time-points, p<0.001; interaction between groups and time, p<0.001), throughout the experiment.

**
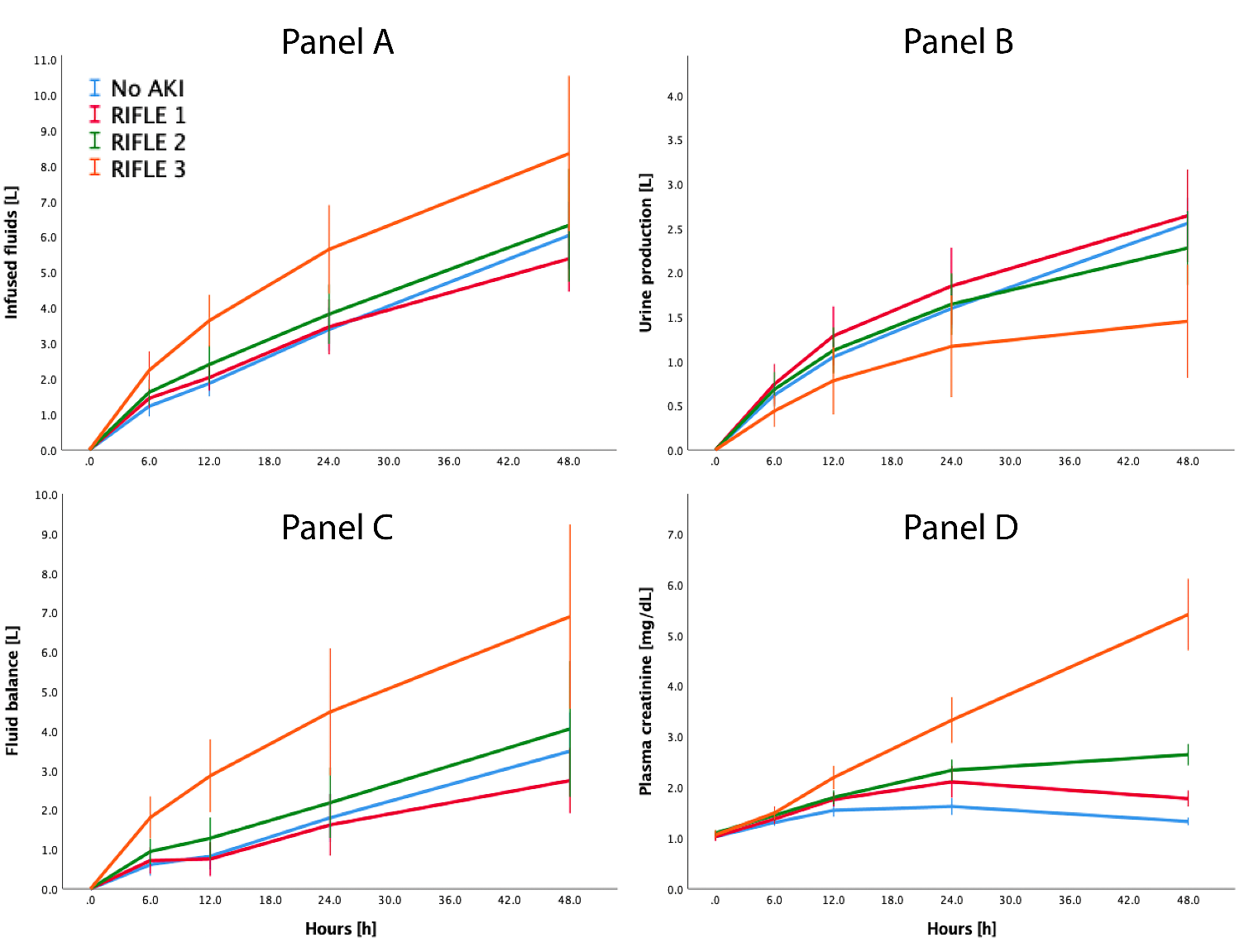
**

**Figure S3:** linear regression model showing the association between plasmatic creatinine collected at 48 hours and mean arterial pressure (Panel A: p<0.001; R^2^ 0.162), mean perfusion pressure (Panel B: p<0.001; R^2^ 0.265) and central venous pressure (Panel C: p=0.004; R^2^ 0.106).


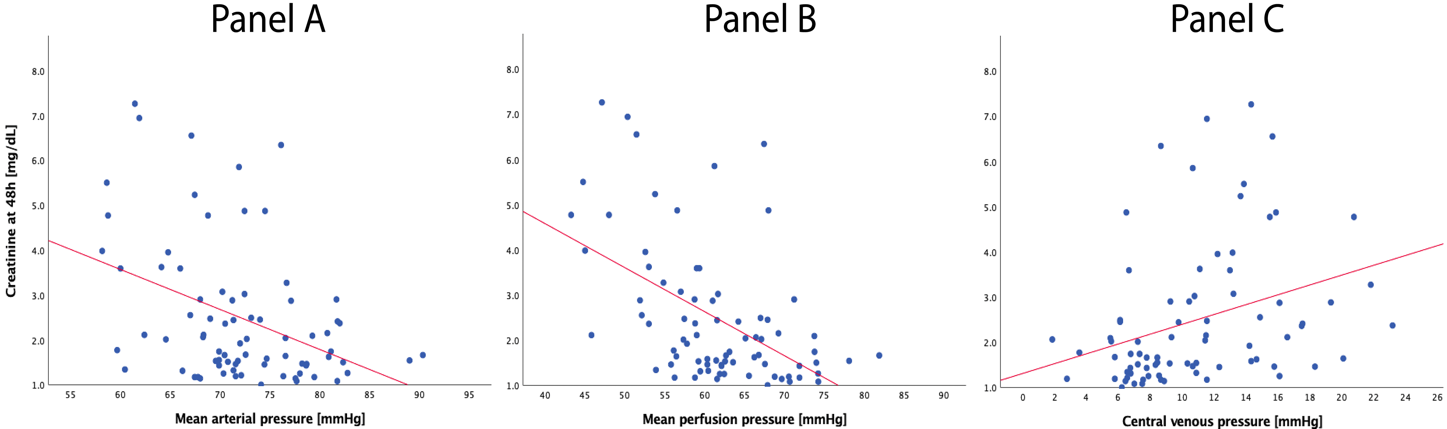


**Figure S4:** Receiver operating characteristic model and area under the curve analysis testing the association between acute kidney injury and hemodynamic variables. Absence of AKI was defined as pertaining to groups: NO AKI and RIFLE 1-Risk; presence of AKI was defined as pertaining to groups RIFLE 2-Injury and RIFLE 3-Failure. MPP showed the highest association (AUC [95%CI] 0.765 [0.657-0.873]), compared to MAP (AUC [95%CI] 0.690 [0.567-0.814]) and CVP (AUC [95%CI] 0.711 [0.591-0.831]).


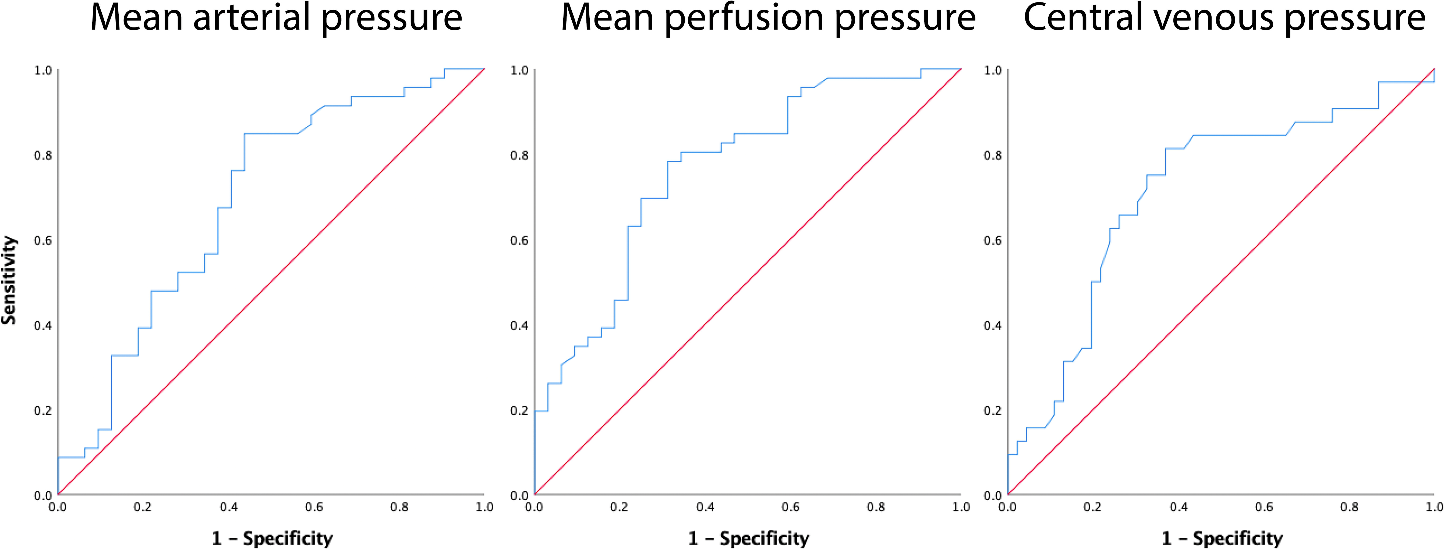


**Figure S5:** linear regression model reporting the association between kidneys’ wet-to-dry ratio and mean arterial pressure (Panel A: p=0.049; R^2^ 0.062), mean perfusion pressure (Panel B: p=0.002; R^2^ 0.145) and central venous pressure (Panel C: p=0.023; R^2^ 0.088).


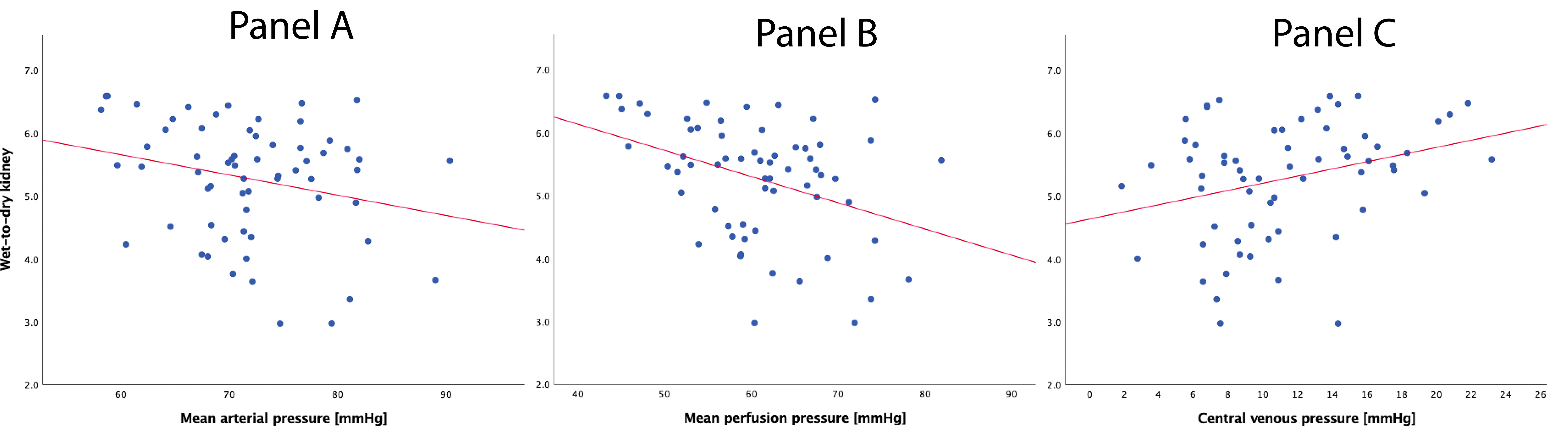


**Figure S6:** linear regression model reporting the association between mean perfusion pressure and PEEP (p<0.001; R^2^ 0.225).


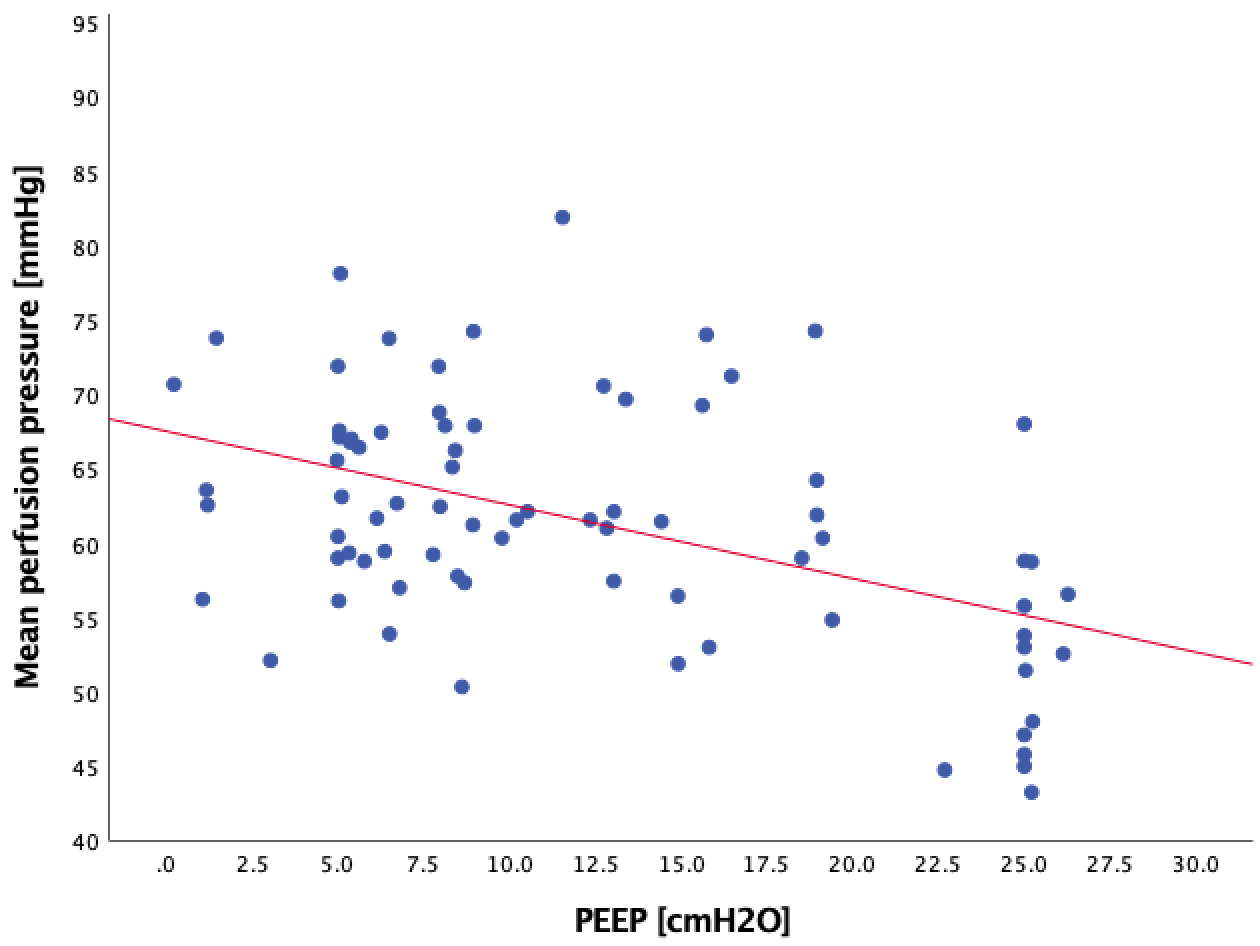


**Figure S7:** linear regression model reporting the association between pleural pressure and PEEP (p<0.001; R^2^ 0.473).

**
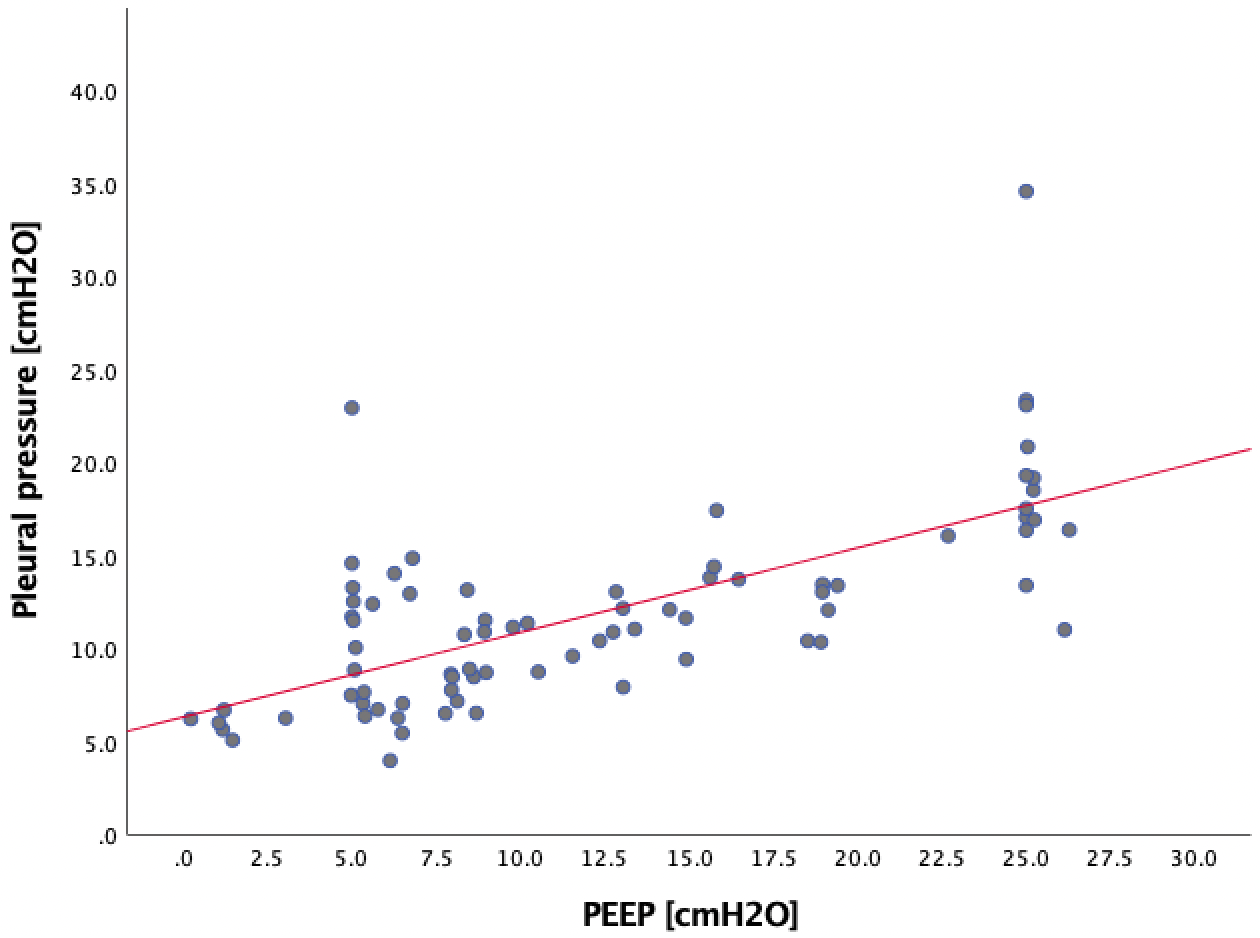
**

**Figure S8. The “vicious circle of fluids”:** schematization of the proposed physiological mechanisms underlying the development of acute kidney injury in ventilated individuals.

High PEEP (1) leads to an increase in pleural pressure (2), that is in turn associated with augmented central venous pressure (CVP) (3). Consequently, the mean perfusion pressure (MPP = MAP - CVP) may be insufficient to ensure adequate renal perfusion, leading to hypoperfusion/congestion of the kidneys (3).

Since increased CVP leads to higher venous return to the heart, if the pump-response is suboptimal, the cardiac output may worsen (4). This would produce a decrease in mean arterial pressure (MAP) (4) and a further impairment of the MPP, eventually leading to a worsen renal congestion/hypoperfusion (5). If any treating physician were to infuse fluids (6), in an attempt to "optimize renal perfusion", the resulting expected increase in CVP (and consequent further reduction in MPP), could generate a dangerous "vicious cycle" of fluids, associated with worsening renal function.


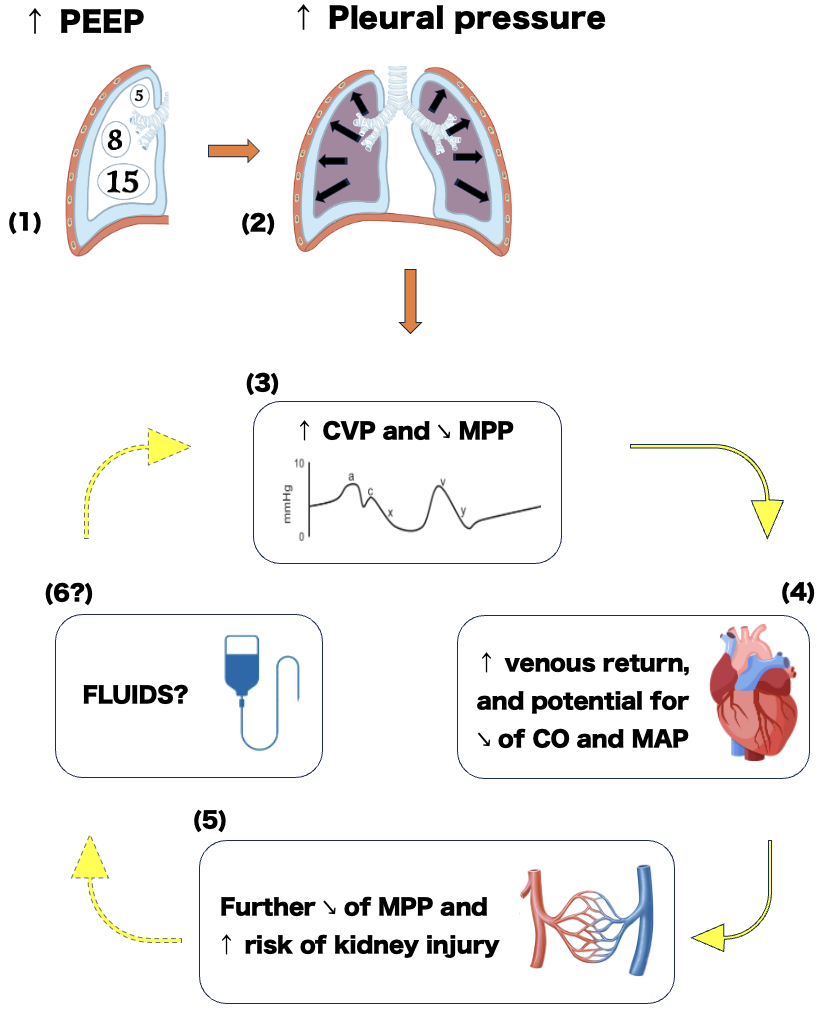


**Supplemental tables**

**Table S1:** A multiple regression model was designed in which the depending variable was the presence of acute kidney injury (absence defined as pertaining to groups: NO AKI and RIFLE 1-Risk; presence defined as pertaining to groups RIFLE 2-Injury) while the independent variable was PEEP. Covariates: tidal volume, respiratory rate and pleural pressure.

| **Variable** | **Odds Ratio** | **95% CI** | **p-value** |
| --- | --- | --- | --- |
| Tidal volume (mL) | 0.001 | -0.001/0.001 | 0.592 |
| Respiratory rate (bpm) | -0.004 | -0.020/0.012 | 0.585 |
| **PEEP (cmH2O)** | **0.035** | **0.007/0.064** | **0.014** |
| Pleural pressure (cmH2O) | -0.021 | 0.063/0.020 | 0.313 |

**Table S2:** Magnitude of the observed effects in the linear mixed-effects model reported in Figure 2.

| **Predictor** | **Estimates** | **Standard error** | **p-value** |
| --- | --- | --- | --- |
| **Pleural pressure** |  |  |  |
| Aki stage | 0.483 | 0.286 | 0.094 |
| **Hour** | **-0.043** | **0.007** | **<0.001** |
| **Interaction** | **0.024** | **0.004** | **<0.001** |
| Conditional R squared 0.688 | | | |
| **PEEP** |  |  |  |
| **Aki stage** | **3.679** | **0.683** | **<0.001** |
| **Hour** | **0.015** | **0.004** | **<0.001** |
| **Interaction** | **-0.010** | **0.002** | **<0.001** |
| Conditional R squared 0.978 | | | |
| **Mean perfusion pressure** |  |  |  |
| **Aki stage** | **-3.680** | **0.884** | **<0.001** |
| **Hour** | **-0.252** | **0.039** | **<0.001** |
| **Interaction** | **-0.010** | **0.024** | **<0.001** |
| Conditional R squared 0.393 | | | |

**Table S3:** Magnitude of the observed effects in the linear mixed-effects model reported in Figure S1.

| **Predictor** | **Estimates** | **Standard error** | **p-value** |
| --- | --- | --- | --- |
| **Mean arterial pressure** |  |  |  |
| **Aki stage** | **-2.888** | **0.830** | **<0.001** |
| **Hour** | **-0.234** | **0.039** | **<0.001** |
| Interaction | 0.009 | 0.024 | 0.691 |
| Conditional R squared 0.302 | | | |
| **Mean perfusion pressure** |  |  |  |
| **Aki stage** | **-3.680** | **0.884** | **<0.001** |
| **Hour** | **-0.252** | **0.039** | **<0.001** |
| **Interaction** | **-0.010** | **0.024** | **<0.001** |
| Conditional R squared 0.393 | | | |
| **Central venous pressure** |  |  |  |
| **Aki stage** | **0.913** | **0.455** | **0.047** |
| **Hour** | **0.020** | **0.008** | **0.012** |
| **Interaction** | **0.021** | **0.005** | **<0.001** |
| Conditional R squared 0.818 | | | |
| **Cardiac output** |  |  |  |
| Aki stage | -0.029 | 0.094 | 0.757 |
| **Hour** | **-0.020** | **0.003** | **<0.001** |
| **Interaction** | **0.011** | **0.001** | **<0.001** |
| Conditional R squared 0.508 | | | |

**Table S4:** Magnitude of the observed effects in the linear mixed-effects model reported in Figure S2.

| **Predictor** | **Estimates** | **Standard error** | **p-value** |
| --- | --- | --- | --- |
| **Fluid infusion** |  |  |  |
| **Aki stage** | **0.352** | **0.177** | **0.049** |
| **Hour** | **0.105** | **0.006** | **<0.001** |
| **Interaction** | **0.007** | **0.003** | **0.073** |
| Conditional R squared 0.778 | | | |
| **Urine production** |  |  |  |
| Aki stage | 0.009 | 0.070 | 0.888 |
| **Hour** | **0.046** | **0.002** | **<0.001** |
| **Interaction** | **-0.006** | **0.001** | **<0.001** |
| Conditional R squared 0.815 | | | |
| **Fluid balance** |  |  |  |
| Aki stage | 0.341 | 0.191 | 0.076 |
| **Hour** | **0.058** | **0.007** | **<0.001** |
| **Interaction** | **0.0.14** | **0.004** | **<0.001** |
| Conditional R squared 0.712 | | | |
| **Creatinine** |  |  |  |
| **Aki stage** | **-0.115** | **0.057** | **0.044** |
| **Hour** | **-0.008** | **0.002** | **<0.001** |
| **Interaction** | **0.025** | **0.001** | **<0.001** |
| Conditional R squared 0.760 | | | |

**Table S5:** Relationship between the AKI status (in the present model the “NO AKI group” was defined as: no AKI + RIFLE 1-Risk; while the “AKI group” was defined as: RIFLE 2-Injury + RIFLE 3-Risk) and PEEP (high PEEP vs. low. PEEP; median of PEEP: 8.965); and between the AKI status and the ratio between chest-wall and respiratory system elastances (high E.cw/E.rs vs. low E.cw/E.rs; median of E.cw/E.rs: 0.392).

|  | **No AKI group** | **AKI2 + AKI 3** | **p-value** |
| --- | --- | --- | --- |
| **PEEP lower than median, n(%)** | **27 (58.7%)** | **10 (31.2%)** | **0.022** |
| **PEEP higher than median, n(%)** | **19 (41.3%)** | **22 (68.8%)** | **0.022** |
| E.cw/E.rs lower than median, n(%) | 19 (41.3%) | 19 (59.4%) | 0.439 |
| E.cw/E.rs higher than median, n(%) | 27 (58.7%) | 13 (40.6%) | 0.439 |
